# Impacts of Adiposity on the Attentional Cost of Sensory-Motor Performance Associated with Mobility in a Dual-Task Paradigm

**DOI:** 10.3390/ijerph192013118

**Published:** 2022-10-12

**Authors:** Abdul Rahim Shaik, Mazen Al Qahtani, Fuzail Ahmad, Mohammad Abu Shaphe, Ahmad H. Alghadir, Anas Alduhishy, Sultan Mofreh A. Assiri, Mohammad Rehan Asad, Amir Iqbal

**Affiliations:** 1 Department of Physical Therapy and Health Rehabilitation, College of Applied Medical Sciences, Majmaah University, Al-Majmaah 11952, Saudi Arabia; 2 College of Applied Sciences, AL Mareefa University, Ad Diriyah 13713, Saudi Arabia; 3 Department of Physical Therapy, College of Applied Medical Sciences, Jazan University, Jazan 45142, Saudi Arabia; 4 Department of Rehabilitation Sciences, College of Applied Medical Sciences, King Saud University, Riyadh 11433, Saudi Arabia; 5 Department of Physical Therapy, College of Applied Medical Sciences, Imam Abdulrahman Bin Faisal University, Dammam 31441, Saudi Arabia; 6 Department of Physical Therapy, Muhayel General Hospital, Asir Health Affairs, Ministry of Health, Abha 62523, Saudi Arabia; 7 Department of Basic Sciences, College of Medicine, Majmaah University, Al-Majmaah 15341, Saudi Arabia

**Keywords:** dual-task paradigm, gait speed, balance, obesity, obese students, BMI

## Abstract

(1) Background: Obesity is one of the most prevalent health problems worldwide. Studies have evidenced that the increase in body weight affects the normal neuromusculoskeletal function, which leads to abnormal gait patterns and impaired balance. (2) Objective: The aim of this study was to examine the influence of dual-task activity (cognitive-motor task) on gait parameters and balance among obese students. (3) Material and methods: A cross-sectional study was conducted among students (18–28 years old), including 120 obese and 120 age-matched normal-weight control subjects, selected at random using simple random sampling, from the Majmaah, Riyadh, Dammam, and Jizan regions of Saudi Arabia. The gait speed was measured in seconds while the controls and the obese subjects performed a dual-task activity of walking down a level, well-lit, narrow lane at their own speed, counting backwards from 100 by 4 s. (4) Results: The results of our study suggest a significant difference in the effect of the dual-task paradigm on the gait speed (*t* = 21.05, *p* = 000) of obese participants when compared to their age-matched counterparts. A significant correlation was found between BMI and gait speed and balance, irrespective of the gender of the obese student. A high degree of positive correlation (r = 0.705, *p* < 0.001) was found between BMI and gait speed, and a high degree of inverse correlation (r = −0.648, *p* < 0.001) was found between BMI and balance among obese students A multiple regression model explained 60% of the variance in gait speed and was statistically significant (R^2^ = 0.60, F (4, 235) = 90.65, *p* = 0.000) with BMI (β = 0.018, *p* = 0.000) and balance (β = 0.015, *p* = 0.000) significantly predicting gait speed. (5) Conclusion: The results of the current study provide evidence that obesity significantly influences gait speed and balance due to the inclusion of a contemporaneous cognitive task. The results also suggest that the dual-task paradigm affects both genders equally.

## 1. Introduction

Obesity is a multifaceted problem that impacts people of all ages. It is caused by a confluence of factors spanning the spectrum from genetics and development to environmental factors [1]. According to the WHO, in the year 2016, around 13% of the world’s adult population was classified as obese, with an obesity rate of 39% among persons aged 18 and older. In what was once thought to be solely an issue affecting wealthy nations, overweight and obesity are now on the rise in low- and middle-income countries, particularly in metropolitan areas [2]. Saudi Arabia (SA) is among the top seven countries with the most significant increases in the rate of obesity at 21.7%, with a comparatively higher female obesity rate [3].

Obesity can impair posture and balance control and can have harmful structural and functional effects on the body. An obese person’s stride is longer and slower than that of a normal-weight person [4]. The aging process deteriorates the gait velocity in independent, community-dwelling, healthy individuals. Also, there is a gender difference in terms of gait speed, not only with increasing age, but also at younger ages [5].

It has been observed that safe walking requires an outstanding level of mobility and cognitive flexibility to regulate movement direction, identify and track visual targets, and also read or communicate. “Attentional focus” refers to a person’s restricted capacity to absorb information to do activities that burden the central nervous system (CNS), limiting the number of tasks they can simultaneously perform [6]. Routine mobility during daily activities necessitates the execution of a contemporaneous cognitive or motor task, such as speaking or holding an object while walking. It has been discovered that concurrently engaging in the second task (i.e., cognitive-motor task) deteriorates the gait speed and balance [7]. When presented with dual-task restrictions, obese children alter their lateral displacement and force allocation, which may impair their capacity to remain safe when multitasking [8]. Gait speed in older people is significantly reduced when performing two tasks at the same time, with greater complexity resulting in greater decrements. In the examination of older adults, this has repercussions, such that a dual-tasking situation can expose vulnerabilities that were not previously apparent during a single-task assessment [9].

The existing literature provides evidence for a strong link between obesity and balance impairments. The gait pattern changes due to these impairments do not, however, substantially vary from normal-weight persons in terms of functional gait stability, especially among young obese adults while they were walking on a level surface [10]. The allocation of cognitive/attentional resources during postural or motor activities has been extensively studied in individuals with pathologies, such as spinal cord injuries or strokes, but this subject does not appear to have been addressed conclusively in the obese population. Stability-related spatiotemporal gait measures, such as step width and the double support time, were not routinely recorded. Such metrics may be able to show the impact of dual-task conditions before gait speed is affected, especially over short distances [11].

When balance disruptions occur, a more complicated biomechanical and cognitive framework is required in everyday life. Walking with a reduced base of support appears to be a suitable compromise as it increases the challenge of keeping a steady posture while also allowing for a parallel assessment of the required attentional demands. Cognitive resources given to control ambulatory processes in obese individuals should be evaluated to understand the cause of gait abnormalities better and optimize therapeutic intervention.

In the present study, we set out to determine the attentional toll obesity takes by utilizing dual-task paradigms (cognitive-motor tasks), in which a cognitive activity is performed at the same time while walking (motor activity) on a narrow path, thereby increasing the difficulty of the task and the attentional cost necessary to maintain it, allowing for the early identification of associated impairments. Therefore, the purpose of this study is to assess the impact of obesity on the attentional cost of sensory-motor performance in terms of gait speed in a dual-task paradigm among college students. The second objective is to examine how gender, age, balance score, and BMI affect gait speed in a dual-task paradigm.

## 2. Materials and Methods

### 2.1. Study Design

A cross-sectional observational study design was selected using simple random sampling for recruitment.

### 2.2. Ethical Consideration

This study obtained ethical approval from the Ethics Sub-Committee of King Saud University (file ID: RRC-2019-02 dated 11 January 2019). The study followed the ethical guidelines for conducting research and preserving the rights of humans involved in the research as in the Declaration of Helsinki (2010). A written, signed, informed consent was obtained from all of the participants.

### 2.3. Sample Size

A statistical application (G*Power 3.1.9.6) used an a priori unpaired *t*-test (two-tailed) to compare the difference between two independent means, keeping a statistical power of 0.5 and an alfa error probability of 0.05. For a medium effect size, the sample was calculated to require 105 in each group. Keeping in view a 10–15% dropout rate, the necessary sample size was calculated to be 120 participants in each group.

### 2.4. Study Participants, Setting, and Sampling

The study was conducted during the year 2019–2020, and a total of 240 students (mean age, 22.72 ± 3.08; 111 males and 129 females), associated with around 80 colleges at various universities in the Riyadh, Dammam, Jizan, and Majmaah regions of Saudi Arabia, were recruited. The inclusion criteria included university students between the ages of 19–28 years, obtaining, at minimum, a score of 24 (or more) on the Mini-Mental State Examination scale (MMSE) [12], and obtaining a score of 45 or above on the Berg Balance Scale (BBS) [13]. The subjects were categorized into groups based on their BMIs. Subjects with a BMI in the range of 18.5–24.9 kg/m^2^ were allocated to the normal weight group, whereas subjects having a BMI ≥ 30 kg/m^2^ were allocated to the obese group [13]. Those subjects with any orthopedic, cardiovascular, neurological, or cognitive impairment, a fear of falling, or a history of having fallen in the previous 12 months were excluded from the study.

### 2.5. Procedures

The participants in both groups walked on a narrow surface, with and without a dual task. Using the distance between the participant’s anterior superior iliac spine, the path’s width was adjusted to 50% of this distance to generate a similar challenge for persons of diverse body topologies [14]. Using a chalk piece, the floor was demarcated, and individuals were encouraged to stay within the designated area. The beginning and the end of a 10-m walkway was designated by chalk makings. All individuals were evaluated while walking barefoot on a 10-m path, with and without a dual task at their own pace. The subjects underwent three trials, and the maximum value among the three trials was regarded as the subject’s gait speed. A digital stopwatch was used to measure the time taken to complete the 10-m distance [15]. To avoid familiarization, participants were randomly assigned to either the simple walking or walking under a dual-task conditions.

All of the participants performed the dual task (i.e., to focus on both motor and cognitive tasks at the same time) activity by walking down a level, well-lit, narrow lane at their own speeds. While walking on a narrow path, which in itself is an attention-demanding task, the subjects were required to complete the cognitive task of counting backward from 100, in increments of 4 [14].

### 2.6. Statistical Methods

An independent *t*-test was performed after verifying for parametric assumptions to compare gait speed (10-MWT) under a cognitively taxing dual-task paradigm between both groups. The Pearson correlation test was used to analyze the association between BMI, gait speed, and balance. The influence of exploratory factors (gender, age, BMI, and balance) on gait speed was estimated using a multiple regression analysis. IBM SPSS Statistics v. 25 was used for all analyses (IBM Corp., Released in 2017, IBM SPSS Statistics for Windows, version 25.0. Armonk, NY, USA).

## 3. Results

In the obese group, 45% of the participants were male, and 55% were female, and similarly, in the control group, 47.5% of the participants were male and 52.5% were female. The mean age of the obese subjects was 22.51 ± 2.95 years, and that of the control subjects was 22.93 ± 3.21 years. The mean height in centimeters (cm) and weight in kilograms (kg) in the obese group was 1.65 ± 0.05 cm and 80.17 ± 4.4 kg, and in the control group, these were 1.67 ± 0.06 cm and 63.04 ± 5.09 kg, respectively. Accordingly, the BMI of the obese group was 29.3 ± 1.5, and that of the age-matched control group was 22.66 ± 0.9.

The gate speed was assessed by a 10-Meter Walk Test, which is the time (in seconds) required to complete a 10 m walking distance at a steady pace. The gate speed is calculated by dividing the 10 m walking distance at a steady pace by the time taken for completion. The average walking speeds of the normal weight and obese groups during the simple walking task were 1.04 ± 0.15 m/s and 0.79 ± 0.11 m/s, respectively, whereas, under the dual-task condition, the average walking speeds of the normal-weight and obese groups were 0.95 ± 0.1 m/s and 0.7 ± 0.08 m/s, respectively.

Table 1 depicts the influence of the sensory-motor dual task on gait speed in obese and normal-weight persons. The gait speed of the obese group was 36% lower than that of the normal group, which was found to be statistically significant (*t* = 21.05, *p* = 000).

The attentional cost of the sensory-motor dual task on gait speed was calculated using the formula ((dual task-simple task)/simple task×100). When the groups were compared, it was discovered that the obese group had an average mean drop of 13% in gait speed during the dual-task condition, resulting in a 9.9% change in the attentional cost of walking, which was statistically significant (*t* = −2.02, *p* = 045).

Table 2 shows the correlation matrix of BMI, gait speed, and balance. The relationships between BMI and gait speed (r = −0.223, *p* = 0.043) and with balance (r = −0.286, *p* = 0.031) were found to be small and negative among control group students (those of a normal weight). There was a statistically significant, moderately negative correlation between gait speed and balance (r = −0.434, *p* = 0.023) found in the obese group, but in the control group, it was found to be non-significant (*p* = 0.948). This evidenced that BMI shows more influence on gait speed and balance among the obese group of participants.

Table 3 presents the effect of exploratory variables (gender, age, BMI, and balance) on gait speed using a multiple linear regression analysis. Multiple linear regression was used to test whether gender, age, BMI, or balance significantly predicted gait speed.

The overall regression model explains 60% of the variation in the gait speed and was statistically significant (R^2^ = 0.60, F (4, 235) = 90.65, *p* = 0.000). It was found that BMI (β = −0.018, *p* = 0.000) and balance (β = 0.015, *p* = 0.000) significantly predicted gait speed, but gender (β = −0.016, *p* = 0.223) and age (β = −0.002, *p* = 0.35) did not significantly predict gait speed.

## 4. Discussion

The aim of this study was to evaluate the impact of a dual-task activity (cognitive-motor task) on gait speed among obese and normal-weight students. The results of our study suggest a significant difference in the effect of the dual-task paradigm on the gait speed of obese participants when compared to their age-matched, non-obese counterparts. A significant correlation was found between BMI, gait speed, and balance, irrespective of the gender of the obese students. A high degree of negative correlation was found between BMI and gait speed and balance among obese students. A regression analysis demonstrated that 60% of the total changes observed in gait speed were due to the exploratory variables, whereas an increase of each unit in BMI caused a change of 0.29 m/s in the gait speed, and a change of one BBS score caused a decrease of 0.22 m/s in gait speed.

A previous study has shown that the accomplishment of a dual task did not significantly affect gait variability among young adults in any of the settings in which they were tested [16]. While dual-tasking does not have any therapeutic implications in healthy young individuals, it has a higher impact on those with disabilities whose gait speed or cognitive tasks necessitate a greater level of attentional demands [17]. In some cases, this may be extremely relevant, such as when individuals may pay more attention to “external interferences” than they do to their own motion [18,19]. Mobility in daily life frequently necessitates the accomplishment of a contemporaneous cognitive or motor task, such as conversing or manipulating things while walking [15], which may affect the speed and safety of ambulation. Several personal attributes have been linked to behaviors that compromise physical ability and cause lower mobility, particularly in children and adolescents [9]. It has been suggested that complex walking tasks, such as dual-task walking, may be more sensitive than leisurely strolling activities for detecting early deficits in postural stability in healthy persons who do not have any functional limitations [20].

Because there was no prioritization of tasks in the current investigation and the individuals were allowed to make mistakes, it is possible that the results were influenced by such a factor [21]. Inaccurate estimations of one’s physical limitations may lead to an incorrect assessment of environmental risks and an increased risk of falling and accidents [22]. However, the ability to multitask might be utilized as a performance-based criterion to identify people with a higher fall risk.

While walking in a difficult environment, a person’s capacity to make appropriate judgments may be challenged by inefficient, incorrect, or even dangerous options, which may result in a person losing their way or squandering time or effort. A narrow base with a dual goal may allow one to disregard extraneous sensory inputs, block out diversions to find solutions, and react accordingly to key environmental factors [14].

### Limitations of the Study

In our study, we did not measure waist–hip circumference, which may have enabled us to make more precise obesity classifications. Further studies can be carried out on individuals with gait impairments, evaluating the effect of muscle power and endurance on task prioritization, which may produce interesting findings.

## 5. Conclusions

The present study’s findings show that obesity, as measured by BMI and balance score, has a substantial impact on gait speed when subjects are walking on a narrow path, owing to the inclusion of a concurrent cognitive activity that places an increased attentional burden on the sensory-motor system.

## Figures and Tables

**Table 1:** Attentional cost on gait speed under a sensory-motor dual-task condition among normal-weight and obese college-going populations.

Sl. No.	Characteristics	Normal-Weight Group (*n* = 120)	Obese Group (*n* = 120)	Unpaired *t*-Test	
		Mean ± SD	Mean ± SD	*t*-Value	*p*-Value
1	Gait speed (m/s)	0.95 ± 0.1	0.7 ± 0.08	21.05	0.000 *
2	Attentional cost of walking (% change)	6.6 ± 14.7	9.9 ± 10.1	−2.02	0.045 *

* -statistically significant (*p* < 0.05).

**Table 2:** Correlation matrix of BMI, gait speed, and balance (Pearson’s r).

Sl. No.	Characteristics	Control (*n* = 120)			Obese (*n* = 120)		
		BMI	Gait Speed	Balance	BMI	Gait Speed	Balance
1	BMI	1.000			1.000		
2	Gait Speed	−0.223 *	1.000		−0.705 **	1.000	
3	Balance	−0.286 *	−0.072	1.000	−0.648 **	−0.434 **	1.000

* -significant (*p* < 0.05); ** -highly significant (*p* < 0.01).

**Table 3:** Multiple linear regression to estimate the effect of exploratory variables (gender, age, BMI, and balance) on gait speed.

Regression Analysis	B	SE	t	*p*	95% CI	
					LB	UB
(Constant)	0.521	0.229	2.272	0.024	0.069	0.079
Gender	−0.14	0.178	−0.785	0.433	−0.01	−0.041
Age	−0.023	0.033	−0.685	0.494	−0.002	0.007
BMI	0.290	0.049	5.90	0.000	−0.024	−0.011
Balance	−0.221	0.042	−5.31	0.000	0.009	0.021

Dependent Variable: Gait Speed in m/s.

## Data Availability

The data set for the result of this study will be available from the corresponding author upon reasonable request.
